# Comparison of Visual Neuroadaptations After Multifocal and Monofocal Intraocular Lens Implantation

**DOI:** 10.3389/fnins.2021.648863

**Published:** 2021-06-14

**Authors:** Li Zhang, Duoru Lin, Yong Wang, Wan Chen, Wei Xiao, Yi Xiang, Yi Zhu, Chuan Chen, Xiying Dong, Yizhi Liu, Weirong Chen, Haotian Lin

**Affiliations:** ^1^State Key Laboratory of Ophthalmology, Zhongshan Ophthalmic Center, Sun Yat-sen University, Guangzhou, China; ^2^Department of Ophthalmology, The Central Hospital of Wuhan, Tongji Medical College, Huazhong University of Science and Technology, Wuhan, China; ^3^Wuhan Aier Eye Hospital, Aier Eye Hospital of Wuhan University, Wuhan, China; ^4^Department of Molecular and Cellular Pharmacology, University of Miami Miller School of Medicine, Miami, FL, United States; ^5^Sylvester Comprehensive Cancer Center, University of Miami Miller School of Medicine, Miami, FL, United States; ^6^Chinese Academy of Medical Sciences, Peking Union Medical College, Beijing, China

**Keywords:** visual disturbances, visual function, visual neuroadaptation, functional magnetic resonance imaging, multifocal intraocular lens, monofocal intraocular lens

## Abstract

Visual neuroadaptation is believed to play an important role in determining the final visual outcomes following intraocular lens (IOL) implantation. To investigate visual neuroadaptation in patients with age-related cataracts (ARCs) after phacoemulsification with multifocal and monofocal IOL implantation, we conducted a prospective, controlled clinical trial in Zhongshan Ophthalmology Center. This study included 22 patients with bilateral ARCs: 11 patients underwent phacoemulsification and multifocal IOL (Mu-IOL) implantation, and 11 patients underwent phacoemulsification and monofocal IOL (Mo-IOL) implantation. Visual disturbances (glare and halos), visual function (including visual acuity, retinal straylight, contrast sensitivity, and visual evoked potentials) and visual cortical function (fractional amplitude of low-frequency fluctuations, fALFF) in Bowman’s areas 17–19 as the region of interest were assessed before and after surgeries. The results showed that the fALFF values of the visual cortex in the Mu-IOL group decreased at 1 week postoperatively and recovered to baseline at 3 months and then improved at 6 months, compared with preoperative levels (at a whole-brain threshold of *P* < 0.05, AlphaSim-corrected, voxels > 228, repeated measures analysis of variance). Significantly increased fALFF values in the visual cortex were detected 1 week after surgery in the Mo-IOL group and decreased to baseline at 3 and 6 months. The fALFF of the lingual gyrus was negatively correlated with visual disturbances (*P* < 0.05). To conclude, early postoperative visual neuroadaptation was detected in the Mu-IOL group by resting-state fMRI analysis. The different changing trends of postoperative fALFF values in the two groups indicated distinct neuroadaptations patterns after Mu-IOL and Mo-IOL implantation.

## Introduction

Cataract extraction and intraocular lens (IOL) implantation are standard treatments for visually impaired age-related cataracts (ARCs). Surgeries can significantly improve the visual acuity and visual function of cataractous patients (Liu et al., 2017). Furthermore, postoperative improvements in gray matter volumes and hemodynamics in visual- and cognition-related areas of brains were also detected in our previous study (Lin et al., 2018). At present, monofocal IOL (Mo-IOL) and multifocal IOL (Mu-IOL) are two main types of IOL (Calladine et al., 2015). Mo-IOLs provide patients with clear vision in one fixed distance, as the focus of Mo-IOLs cannot be adjusted. Mu-IOLs help reduce the need for spectacles for both near and distance visual tasks (Khandelwal et al., 2019). Although Mu-IOLs are effective at improving near vision, some patients reported lower contrast sensitivity (CS) (Ji et al., 2013) and visual disturbances (Cao et al., 2019), such as glare and halos (Kalantzis et al., 2014). Mu-IOLs cause a dispersion of the energy of the light entering into the eyes by separating light into different foci, which results in a change in the physiology of vision (Alio et al., 2017). A process of neuroadaptation, the capability of the brain to adapt to changes, can be activated after Mu-IOL implantation to adjust the neurophysiology of the changes that are induced in the quality of the retinal image by light dispersion.

Functional magnetic resonance imaging (fMRI) is a non-invasive technique that allows investigations of neuronal activities in the brain (Ogawa et al., 1990). fMRI has been widely used in assessing the brain function of patients with impaired visual systems, such as glaucoma-induced visual loss (Dai et al., 2013) and amblyopia (Wang et al., 2014). Rosa’s group has assessed neuroadaptation to multifocal IOLs by task-based fMRI for the first time. The fractional amplitude of low-frequency fluctuations (fALFF) method was used to investigate the abnormalities of regional brain function activity in patients with oculopathy [glaucoma (Yuan et al., 2018), strabismus (Min et al., 2018), et al.]. Here, we focus on the preoperative and postoperative activities of visual cortical neurons with resting-state fMRI to investigate visual disturbances and visual neuroadaptation in ARC patients after Mu-IOL implantations, and compare them with Mo-IOL implantations.

Both Mo-IOL and Mu-IOL have advantages and disadvantages, and knowing about the differences in visual neuroadaptation between the two types of IOL implantations is beneficial to the understanding of postoperative changes in visual function and brain recovery among patients with different IOL implantations, and has clinical significance for the IOL selection in ARC patients.

## Materials and Methods

### Subjects

**FIGURE 1:**
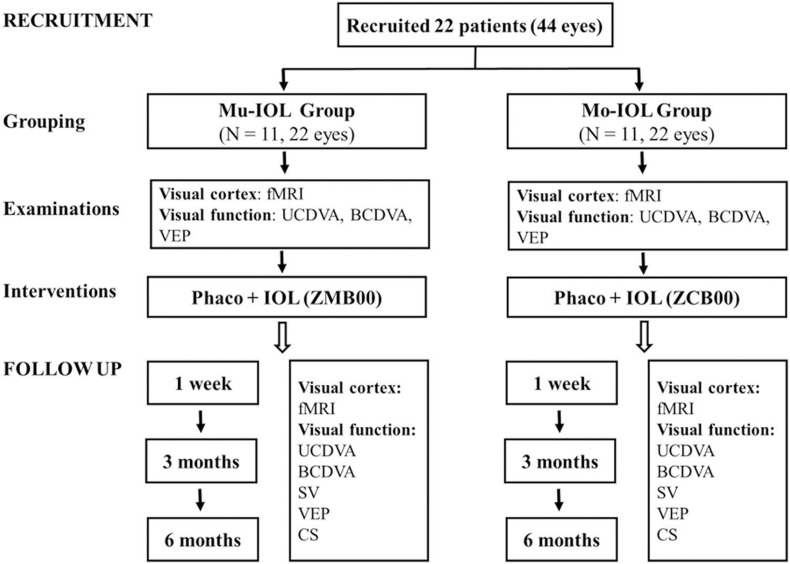
Flowchart of recruitment and follow-up evaluations. fMRI, functional magnetic resonance imaging; UCDVA, uncorrected distance visual acuity; BCDVA, best-corrected distance visual acuity; CS, contrast sensitivity; SV, straylight values; VEP, visual evoked potential; Mo-IOL, monofocal intraocular lens; Mu-IOL, multifocal intraocular lens.

Twenty-two (44 eyes) right-handed patients with ARCs were recruited from the Zhongshan Ophthalmic Center (ZOC), and written informed consent was obtained from all participants or their legal guardians. This trial was registered with ClinicalTrials.gov, NCT02644720. This study was approved by the Ethics Committee of the ZOC at Sun Yat-sen University (2014MEKY035) and adhered to the tenets of the Declaration of Helsinki. The study design was shown in Figure 1, and the patients were allocated to a Mu-IOL group or a Mo-IOL group depending on the patient’s choice in an age-, sex-, and education-matched manner. Eleven patients (22 eyes) were assigned to the Mu-IOL group: Tecnis ZMB00 (AMO, Inc., CA, United States), and 11 patients (22 eyes) were assigned to the Mo-IOL group: Tecnis ZCB00 (AMO, Inc., CA, United States). The inclusion criteria were bilateral cataracts as classified by the Lens Opacity Classification System III, corneal astigmatism of less than 1.0 diopters (D), and IOL power between +18.0 and +25.0 D. Patients with a history of neurological or psychiatric disorder, degenerative optical diseases, associated ocular or systemic diseases, who had prior refractive, glaucoma or penetrating keratectomy surgery, or conditions that could potentially interfere with the final results were excluded. Cataract surgeries were performed by the same surgeon (WRC). The standard technique in all patients consisted of sutureless phacoemulsification using the Legacy 2000 Series and Infinity phacoemulsification machine (Alcon Laboratories Inc., Fort Worth, TX, United States), with clear corneal incisions up to 3.2 and 5.0–5.5 mm capsulorrhexis. Surgery on the fellow eye was performed 1 month later for each patient. Levels of education were evaluated by the cumulative time spent in school.

### Visual Acuity, Retinal Straylight, Contrast Sensitivity, Pattern Visual Evoked Potential, fMRI Examinations and Visual Disturbances

Uncorrected distance visual acuity (UCDVA; logMAR) and best-corrected distance visual acuity (BCDVA; logMAR) were examined with a Standard Logarithmic Visual Acuity E chart (Snellen) preoperatively and at 1week, 3 months, and 6 months postoperatively. The measurement of retinal straylight value (SV) was performed with a C-Quantstraylight meter (Oculus Optikgerate GmbH, Wetzlar, Germany). Contrast sensitivity was tested by Contrast Glare Tester 1000 (CGT-1000 Takagi Seiko Co. Ltd., Nagano, Japan). The pattern visual evoked potential (PVEP) was recorded using an Espion system (Diagnosys LLC, Lowell, MA, United States). The measurements of SV, CS, and PVEP were performed at 1 week, 3 months, and 6 months postoperatively (after the second eye). Resting-state fMRI acquisitions were performed on a MAGNETOM Verio 3T MR scanner (A Tim System; Siemens, Erlangen, Germany) preoperatively and at postoperative intervals of 1 week, 3 months, and 6 months. The details of examinations were all showed in Supplementary Materials.

This study focused on patients’ perceived severity of two major symptoms, glare and halos, as a proxy of their subjective visual disturbances. Patients rated their perception of glare or halos on four subscales (4 = severe; 3 = moderate; 2 = mild; and 1 = none). The final scores on the overall level of visual disturbances were determined by taking the mean of the ratings on “glare” and “halos.” Assessments were made at postoperative intervals of 1 week, 3 months, and 6 months postoperatively.

### Data Preprocessing and fALFF Analysis

Standard professional data processing software, Data Processing Assistant for Resting-State fMRI (DPARSF 2.2; State Key Laboratory of Cognitive Neuroscience and Learning, Beijing Normal University, Beijing, China; available in the public domain at http://rfmri.org/DPARSF), was used. The Resting-State fMRI Data Analysis Toolkit^1^ was used to calculate fALFF (Supplementary Materials). The fALFF was used as a normalized index of ALFF by providing the relative amplitude of the low-frequency domain against the entire spectrum of frequencies, which represented the spontaneous neuronal activity of the brain. The visual cortex mainly includes BA17, BA18, and BA19 (Waberski et al., 2008). BA17 is defined as the primary visual area (PVA), which is directly connected with retinal ganglion cells (RGCs) (Audoin et al., 2006). BA18 and BA19 are defined as higher visual cortices that receive input information from the PVA. Therefore, we mainly focused on these Brodmann areas (BA17, BA18, and BA19) as the region of interest (ROI) when visual cortical function (fALFF values) was evaluated.

### Statistical Analysis

The data were entered into the Statistical Package for the Social Sciences (SPSS ver. 24.0, Chicago, IL, United States). A mixed-design analysis of variance (ANOVA) (Lin et al., 2018) was performed to evaluate the differences in visual function between the Mu- and Mo-IOL groups (transverse comparison between different groups) and the changes (longitudinal comparison within groups) before and after surgery. We compared the impact of the specific type of implanted IOL (Mu- or Mo-IOL) on the subjects’ visual quality, i.e., visual quality. perceived level of visual disturbances by independent sample *t*-tests (between different groups) or repeated-measures analysis of variance (within groups). Two-sample *t*-tests were performed to examine the differences in fALFF values between the Mu- and Mo-IOL groups. Repeated-measures ANOVA was used to compare fALFF values in the visual cortex under preoperative and postoperative conditions. The resulting statistical map was set at a combined threshold of a *P* value < 0.05, with a minimum cluster size of 228 mm^3^, corresponding to a corrected threshold of *P* < 0.05 as determined by AlphaSim. We also performed a correlation analysis to determine whether the three variables, namely, visual function, visual disturbances, and visual cortical function (fALFF values), were related.

## Results

### Demographics

This prospective study included 44 eyes of 22 patients (10 males and 12 females) in an age range of 50–72 years (65.18 ± 5.86 years). The mean ages of the 11 patients (five males and six females) in the Mu- and Mo-IOL groups were 65.27 ± 6.02 and 65.09 ± 5.99 years, respectively. The patients in the two groups received the same level of education (Mu-IOL vs Mo-IOL: 11.27 ± 4.36 vs 11.18 ± 4.71 years, *P* = 0.963).

### Visual Function Assessment

In the Mu-IOL group, as shown in Table 1, both UCDVA and BCDVA were improved after surgery. The retinal SV of log(s) was 1.54 ± 0.13 at 1 week postoperatively and significantly decreased at 3 months (1.32 ± 0.15) and 6 months (1.26 ± 0.11). The log(CS) values observed at 3 or 6 months postoperatively were significantly higher than those at 1 week. Additionally, the postoperative peak time and P100 amplitude were improved compared with preoperative levels. These data indicate that the visual function of patients in the Mu-IOL group was improved after surgery.

**TABLE 1:** Comparison of visual function between the Mu- and Mo-IOL groups.

**Visual function**		**IOL**	**Pre**	**1w**	**3m**	**6m**
VA	UCDVA	Mu-IOL	0.71 ± 0.37	0.02 ± 0.05*	0.02 ± 0.08*	0.03 ± 0.07*
		Mo-IOL	0.94 ± 0.39	0.08 ± 0.09*	0.03 ± 0.08*	0.03 ± 0.08*
		*P*	0.057	0.132	0.538	0.821
	BCDVA	Mu-IOL	0.55 ± 0.35	−0.03 ± 0.04*	−0.04 ± 0.04*	−0.04 ± 0.04*
		Mo-IOL	0.68 ± 0.37	−0.05 ± 0.04*	−0.05 ± 0.04*	−0.06 ± 0.04*
		*P*	**0.025^#^**	**0.005^#^**	0.134	0.127
VEP	Amplitudes	Mu-IOL	7.62 ± 3.25	10.51 ± 4.47*	10.35 ± 2.51*	9.88 ± 3.31*
		Mo-IOL	8.21 ± 3.07	15.50 ± 4.50*	15.97 ± 4.58*	14.13 ± 3.59*
		*P*	0.538	**0.001^#^**	**0.000^#^**	**0.000^#^**
	Latencies	Mu-IOL	122.81 ± 11.79	109.55 ± 7.68*	108.90 ± 6.03*	107.82 ± 5.13*
		Mo-IOL	121.00 ± 16.32	105.05 ± 6.03*	104.45 ± 4.83*	104.82 ± 4.74*
		*P*	0.674	**0.036^#^**	**0.013^#^**	0.051
SV		Mu-IOL	N/A	1.54 ± 0.13	1.32 ± 0.15**	1.26 ± 0.11**
		Mo-IOL	N/A	1.41 ± 0.16	1.30 ± 0.17**	1.24 ± 0.21**
		*P*	N/A	**0.004^#^**	0.665	0.743
CS	6.3	Mu-IOL	N/A	1.53 ± 0.15	1.57 ± 0.10	1.63 ± 0.09**
		Mo-IOL	N/A	1.57 ± 0.11	1.72 ± 0.12**	1.72 ± 0.14**
		*P*	N/A	0.247	**0.000^#^**	**0.012^#^**
	4	Mu-IOL	N/A	1.34 ± 0.13	1.42 ± 0.14**	1.48 ± 0.11**
		Mo-IOL	N/A	1.43 ± 0.12	1.53 ± 0.09**	1.54 ± 0.13**
		*P*	N/A	**0.014**	**0.003**	**0.085**
	2.5	Mu-IOL	N/A	1.13 ± 0.16	1.21 ± 0.15**	1.24 ± 0.15**
		Mo-IOL	N/A	1.17 ± 0.12	1.29 ± 0.10**	1.35 ± 0.14**
		*P*	N/A	0.417	**0.040^#^**	**0.008^#^**
	1.6	Mu-IOL	N/A	0.88 ± 0.15	1.01 ± 0.13**	1.07 ± 0.15**
		Mo-IOL	N/A	0.91 ± 0.14	1.09 ± 0.22**	1.10 ± 0.23**
		*P*	N/A	0.544	0.157	0.534
	1.0	Mu-IOL	N/A	0.54 ± 0.12	0.66 ± 0.13**	0.68 ± 0.15**
		Mo-IOL	N/A	0.65 ± 0.15	0.73 ± 0.17**	0.78 ± 0.15**
		*P*	N/A	**0.008^#^**	0.103	**0.030^#^**
	0.7	Mu-IOL	N/A	0.36 ± 0.03	0.37 ± 0.05	0.41 ± 0.07**
		Mo-IOL	N/A	0.38 ± 0.08	0.43 ± 0.09**	0.43 ± 0.09**
		*P*	N/A	0.175	**0.004^#^**	0.279

*P < 0.05 compared with Pre.

**P < 0.05 compared with 1w.

*^#^*P* < 0.05 comparing Mu-IOL with Mo-IOL.*

*Pre, preoperative; 1 w, 1 week postoperatively; 3 m, 3 months postoperatively; 6 m, 6 months postoperatively; Mu-IOL, multifocal intraocular lens; Mo-IOL, monofocal intraocular lens; SV, stray light value; CS, contrast sensitivity; UCDVA, uncorrected distance visual acuity; BCDVA, best-corrected distance visual acuity; SD, standard deviation.*

In the Mo-IOL group, both UCDVA and BCDVA were significantly improved after surgery. The retinal SV of log(s) was significantly lower at 6 months (1.24 ± 0.21) postoperatively than that in the 1-week postoperative period (1.41 ± 0.16). The log (CS) values at the follow-ups of 3 and 6 months were significantly higher than those at 1 week postoperatively. The peak time was shortened and the P100 amplitude was increased. These data also indicate a steady postoperative improvement of visual function of patients in the Mo-IOL group.

Better performance of SV, VEP, and CS was found in the Mo-IOL group than in the Mu-IOL group at 1 week and 3 months after surgery. At 6 months, the overall visual function between the two groups was comparable; however, patients with Mo-IOL exhibited a higher P100 amplitude and CS (especially for the high frequency) than those with Mu-IOL.

### Visual Disturbance Assessment

**FIGURE 2:**
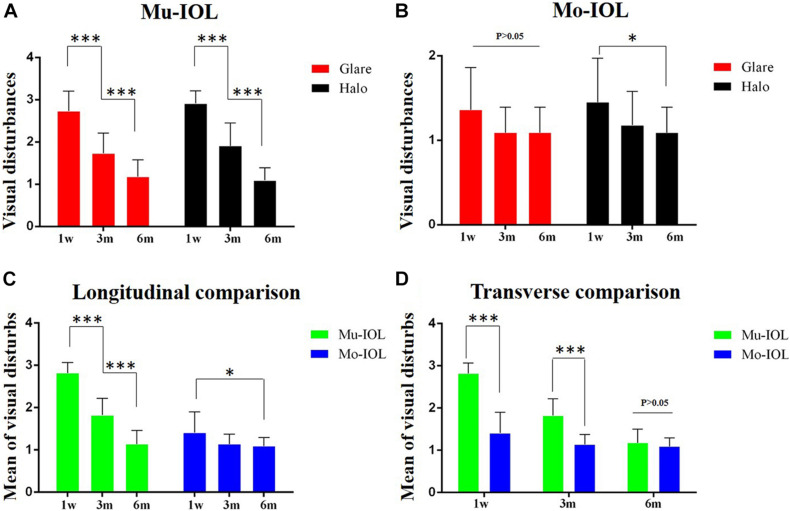
Comparison of visual disturbances between two groups. **(A)** Visual disturbances ratio for glare and halos faded away gradually during the follow-up in the Mu-IOL group; **(B)** Visual disturbances ratio for halos reduced stepwise during the follow-up in the Mo-IOL group; **(C)** The mean of visual disturbances diminished gradually in both Mu-IOL and Mo-IOL groups; **(D)** The mean value in the Mu-IOL group was more severe than in the Mo-IOL group at 1 week or 3 months postoperation. BA, Brodmann area; Mo-IOL, monofocal intraocular lens; Mu-IOL, multifocal intraocular lens; 1 w, 1 week postoperatively; 3 m, 3 months postoperatively; 6 m, 6 months postoperatively; pre, preoperatively (^∗^<0.05; ^∗∗∗^<0.001).

Self-reported glare severity gradually decreased in both the Mu-IOL group (2.73 ± 0.47, 1.73 ± 0.47, and 1.18 ± 0.40) and the Mo-IOL group (1.36 ± 0.50, 1.09 ± 0.30, and 1.09 ± 0.30) at 1 week, 3 months, and 6 months postoperatively. In addition, the inconvenience of halos declined in both the Mu-IOL group (2.91 ± 0.30, 1.91 ± 0.54, and 1.09 ± 0.30) and the Mo-IOL group (1.45 ± 0.52, 1.18 ± 0.40, and 1.09 ± 0.30) at 1 week, 3 months, and 6 months postoperatively. The overall level of visual disturbances, calculated as the average of “glare” and “halos”, was more severe in the Mu-IOL group than in the Mo-IOL group at 1 week (2.82 ± 0.25 vs 1.41 ± 0.49, *P* < 0.001) and 3 months postoperatively (1.82 ± 0.40 vs 1.14 ± 0.23, *P* < 0.001). However, no statistically significant differences were noted between the two groups at 6 months postoperatively (1.14 ± 0.32 vs 1.09 ± 0.20, *P* = 0.452) (Figure 2).

### Preoperative and Postoperative Visual Cortical Function (fALFF Values)

**FIGURE 3:**
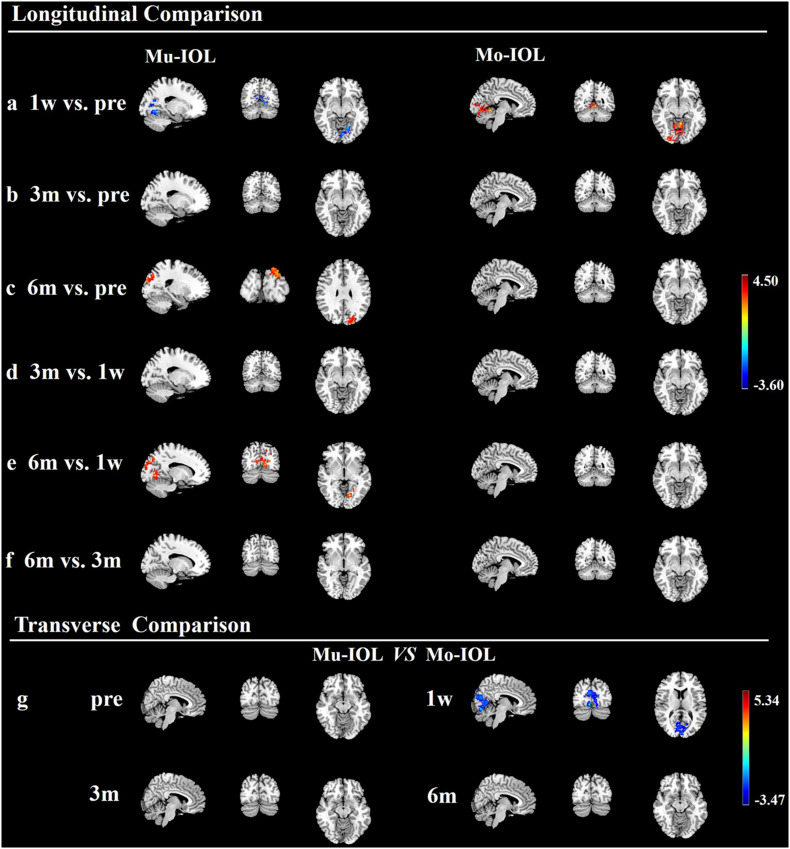
Longitudinal and transverse comparisons of fractional amplitude of low-frequency fluctuations (fALFF) value in two groups. Surface maps show the fALFF value changes in the visual cortex between preoperative and postoperative time points (at a whole-brain threshold of *P* < 0.05, AlphaSim-corrected, voxels >228, repeated measures (ANOVA) or between the Mu- and Mo-IOL groups (at a whole-brain threshold of *P* < 0.05, AlphaSim corrected, voxels >228, two-sample *t*-tests). Slice overlays and plots represent the mean signals from the smoothed difference images for each cluster. Blue and cyan reflect decreases. Red and yellow reflect increases, and *t* indicates the peak *t*-score value for the *t*-test. **(a)** Compared with preoperative values, significantly decreased fALFF values in the Mu-IOL group, but increased values in the Mo-IOL group, in the lingual gyrus (BA17, BA18, and BA19) were observed at 1 week postoperation; **(b)** Compared with preoperative values, no significant difference was found in either the Mu- or the Mo-IOL group at 3 months postoperation; **(c)** Compared with preoperative values, significantly increased fALFF values in the Mu-IOL group in the cuneus (BA18 and BA19), but no significant difference in the Mo-IOL group, were observed at 6 weeks postoperation; **(d)** No significant difference was found in both Mu- and Mo-IOL groups between 1 week and 3 months postoperation; **(e)** Compared with 1-week postoperation value, significantly increased fALFF values in the Mu-IOL group in the lingual gyrus (BA17, BA18, and BA19) but no significant difference in the MO-IOL group was observed 6 weeks after surgery; **(f)** No significant difference was found in either the Mu- or the Mo-IOL group between 3 and 6 months postoperation; **(g)** Comparing the Mo-IOL group to the Mu-IOL group, significantly increased fALFF values in the lingual gyrus (BA17, BA18, and BA19) were reported only at 1 week postoperation. BA, Brodmann area; Mo-IOL, monofocal intraocular lens; Mu-IOL, multifocal intraocular lens; 1 w, 1 week postoperatively; 3 m, 3 months postoperatively; 6 m, 6 months postoperatively; pre, preoperatively.

Compared with preoperative levels, the fALFF values of the visual cortex in the Mu-IOL group decreased at 1 week postoperatively and recovered to baseline at 3 months, and then improved at 6 months postoperatively (at a whole-brain threshold of *P* < 0.05, AlphaSim-corrected, voxels > 228, repeated measures ANOVA) (Figures 3a–c and Table 2). Compared with 1-week postoperation value, no significant difference was found at 3 months postoperation, but significantly increased fALFF values was observed 6 weeks after surgery (Figures 3d,e and Table 2). No significant difference was found between 3 and 6 months postoperation (Figure 3f and Table 2). In the Mo-IOL group, significantly increased fALFF values in the visual cortex were detected 1 week after surgery and decreased to baseline at 3 and 6 months (Figures 3a–c and Table 2).

**TABLE 2:** Comparison of fALFF values (visual cortex) after surgery in the Mu- and Mo-IOL groups.

	**Contrasts**	**Regions**	**BA**	**Cluster size (voxels)**	**MNI coordinates**			***t*-Score for peak voxels**
					***x***	***y***	***z***	
**Longitudinal**	**Mu-IOL**							
**comparison**	1 w vs Pre	Lingual gyrus	17/18/19	405	−18	−60	−9	−4.1499
	3 m vs Pre	N/A	N/A	N/A	N/A	N/A	N/A	N/A
	6 m vs Pre	Cuneus	18/19	244	−21	−93	30	3.7376
	3 m vs 1 w	N/A	N/A	N/A	N/A	N/A	N/A	N/A
	6 m vs 1 w	Lingual gyrus	17/18/19	702	−15	−72	−3	4.6322
	6 m vs 3 m	N/A	N/A	N/A	N/A	N/A	N/A	N/A
	**Mo-IOL**							
	1 w vs Pre	Lingual gyrus	17/18/19	478	−6	−51	−9	4.3132
	3 m vs Pre	N/A	N/A	N/A	N/A	N/A	N/A	N/A
	6 m vs Pre	N/A	N/A	N/A	N/A	N/A	N/A	N/A
	3 m vs 1 w	N/A	N/A	N/A	N/A	N/A	N/A	N/A
	6 m vs 1 w	N/A	N/A	N/A	N/A	N/A	N/A	N/A
	6 m vs 3 m	N/A	N/A	N/A	N/A	N/A	N/A	N/A
**Transverse**	**Mo- vs Mu-IOL**							
**comparison**	Pre	N/A	N/A	N/A	N/A	N/A	N/A	N/A
	1 w	Lingual gyrus	17/18/19	689	6	−78	−12	5.3405
	3 m	N/A	N/A	N/A	N/A	N/A	N/A	N/A
	6 m	N/A	N/A	N/A	N/A	N/A	N/A	N/A

*Repeated measures ANOVA with AlphaSim correction (>228 voxels) was used for the longitudinal comparison between preoperative and postoperative time points in the Mu- or Mo-IOL group in the visual cortex (BA17, 18, and 19). A two-sample *t*-test with AlphaSim correction (>228 voxels) was used for the transverse comparison between the Mu- and Mo-IOL groups (*P* < 0⋅05). A positive peak *t*-score value indicates an increase, and a negative value indicates a decrease. *x, y, z*: MNI coordinates of primary peak locations in the Talairach space.*

*fALFF, fractional amplitudes of low-frequency fluctuations; BA, Brodmann area; MNI, Montreal Neurological Institute; Pre, preoperative; 1 w, 1 week postoperatively; 3 m, 3 months postoperatively; 6 m, 6 months postoperatively; Mo-IOL, monofocal intraocular lens; Mu-IOL, multifocal intraocular lens.*

The fALFF values in the visual cortex of the Mo-IOL group were significantly higher than those of the Mu-IOL group at 1 week after surgery. No statistically significant differences in fALFF values were found at preoperative, 3 month or 6 month assessments between the Mu- and Mo-IOL groups. The different changing trends of postoperative fALFF values in the two groups indicated distinct visual neuroadaptations after Mu-IOL and Mo-IOL implantation (Figure 3g and Table 2).

### Correlation Analysis of Visual Disturbances, Visual Function, and Visual Cortical Function (fALFF Values)

**FIGURE 4:**
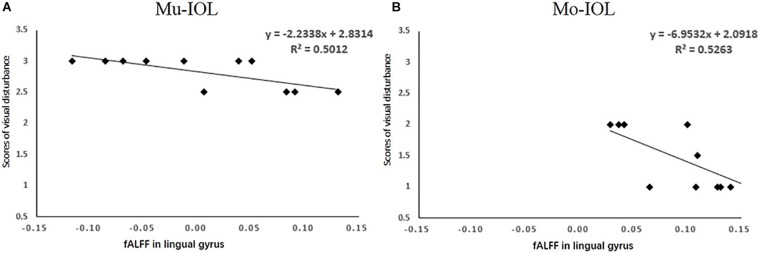
Relationships between the mean of visual disturbances and the fALFF value in the lingual gyrus in the Mu-IOL group **(A)** and in the Mo-IOL group **(B)**. No significant correlations were noted between visual disturbance and visual function. fALFF, the fractional amplitude of low-frequency fluctuations; Mo-IOL, monofocal intraocular lens; Mu-IOL, multifocal intraocular lens.

A correlation analysis was performed among the self-reported level of visual disturbances, visual acuities (BCDVA, UCDVA, SV, CS, and PVEP) and visual cortical functions (fALFF) based on data 1 week after surgery. Only the fALFF of the lingual gyrus for each patient was negatively correlated with the overall level of visual disturbances as rated by the subjects in both the Mu-IOL (Figure 4A, *R*^2^ = 0.50, *P* < 0.05) and Mo-IOL (Figure 4B, *R*^2^ = 0.53, *P* < 0.05) groups. These findings indicated that the level of visual disturbances and visual cortical function were closely related.

## Discussion

Pattern of visual stimulation changes after cataract extraction and IOL implantations, visual neuroadaptation may occur to adapt to these changes, showing an alteration of brain function. Our previous study showed that ocular reconstruction can functionally and structurally reverse cataract-induced brain changes (Lin et al., 2018). However, the difference in functional brain recovery by visual restoration between Mu-IOL and Mo-IOL implantation remains unclear. Comparisons between Mu-IOL and Mo-IOL have been previously studied, and most of them focused on visual outcomes and quality of life evaluated through questionnaires and optical parameters such as CS (Vingolo et al., 2007; Ji et al., 2013), glare perception (Vingolo et al., 2007), wavefront aberrations (Liao et al., 2018) and objective optical qualities (Pedrotti et al., 2018). In this study, improved visual function was found after surgery in both the Mu- and Mo-IOL groups. Furthermore, the patients in the Mu-IOL group exhibited a significant postoperative decline in fALFFs in the visual cortex (lingual gyrus) during the first week and increased thereafter, while patients in the Mo-IOL group showed immediately increased visual cortical function at 1 week after surgery and decreased to baseline at 3 months postoperatively.

In general, the focus of the lens is adjustable according to demand. When people focus on a distant object, the images of objects within a near distance are blurred in the brain, which is the same as the imaging of patients with Mo-IOL implantations. The full diffractive Mu-IOLs in this study provide a clear vision of the whole distance by redistributing thelight energy. The different sharpness and brightness of images in patients after Mu-IOL implantations may induce immediate postoperative visual neuroadaptation.

The lingual gyrus is an important component of the visual attention network involved in the bottom-up attentional pathway, which has been proposed as a circuit-breaker to reorient attention to new external information (Goodale and Milner, 1992; Corbetta et al., 2008; Igelström and Graziano, 2017). The lingual gyrus has a critical function in spatial memory (Sulpizio et al., 2013) and visual attention (Gebodh et al., 2017). The different trends in the two groups suggest that the implantation of different types of IOLs may rewire distinct visual attention networks. Previous studies showed that the adaptation of the dorsal lateral geniculate nucleus to grating stimuli could be changed by activating either GABAA or GABAB receptors, indicating that GABAergic inhibition is responsible for visual adaptation (Yang et al., 2003). Therefore, visual neuroadaptation might be closely related to GABAergic inhibition in patients following the implantation of Mu- or Mo-IOLs.

In the present study, we evaluated the changes in visual functions after surgery in both Mu-IOL and Mo-IOL groups. Studies have shown that visual acuity improves following a properly performed cataract surgery (Liu et al., 2017). The postoperative mean UCDVA (logMAR) and mean BCDVA (logMAR) are higher than the postoperative mean. Retinal straylight impacts visual performance (Van Den Berg et al., 2007; Liu et al., 2017). Previous studies have indicated that no significant differences in SVs (Cerviño et al., 2008) or higher SVs (de Vries et al., 2008) were found at 6 month postoperatively, comparing Mu-IOL with Mo-IOL. However, changes in straylight after surgery with time have rarely been reported in literature. Based on our study of the two groups, the “straylight” parameter values at 3- and 6-month assessments were significantly lower than that at 1 week from the day of one’s surgery. Contrast sensitivity has been taken as an important parameter for visual function in Mu-IOL (Souza et al., 2006; de Vries et al., 2008). A statistically significant increase in CS with time was noted in the postoperative intervals of 6 months, similar to Robert’s results (Montés-Micó and Alió, 2003). Despite of the fact that VEP can also be used for preoperative assessments of visual functions in cataract eyes (Mori et al., 2001), it has rarely been used for in a postoperative setting. Our research showed that latencies of PVEP, when compared to that at the first postoperative week, were lower at 3 and 6 months after surgeries. This decline in PVEP’s latency indicated a recovery of visual function. The process of neuroadaptation is accompanied by the improvement of visual function. Although no significant correlations were indicated between visual function (VA, CS, SV, and PEP) and visual cortical function/disturbances, the improvement of visual function maybe imply the occurrence of neuroadaptation.

Visual disturbances are a visual phenomenon that is irrelevant to visual acuity (Kretz et al., 2016) or visual function (Rosa et al., 2017). Visual disturbances, to some extent, reflect the brain function of the visual cortex (Khammash et al., 2019). In this study, we found that visual disturbances and visual cortical function were closely related. Compared with Mo-IOLs, patients with Mu-IOL implantation showed a higher level of visual disturbances and a reduction in fALFF values at 1 week postoperatively, indicating adaptation suppression in the early postoperative stage. However, it was found that these visual disturbances were greatly improved over time when visual neuroadaptation occurred in the study. Therefore, necessary preoperative communication and adaptability evaluation are clinically significant for patients who want to undergo Mu-IOL implantation. Measures to reduce postoperative visual disturbances, such as improving the design and industrial manufacture of Mu-IOLs, may help to shorten visual neuroadaptation.

Several limitations of the study should be considered when interpreting the results. First, only three time points of postoperative follow-up (1 week, 3 months, and 6 months) were included in the study design, and the detailed adaptive progress and the turning points from a decrease (the lowest point) to an increase of fALFF in patients with Mu-IOL implantation remain unclear. Second, visual neuroadaptation for different types of Mu-IOLs (diffractive vs refractive) still needs further investigation. Third, further studies are required to uncover the molecular and cellular mechanisms of visual neuroadaptation following cataract surgery. A multicenter randomized controlled clinical trial that received different types of IOL implantations would be ideal for the results to be representative of a general population and to minimize their bias and confounding factors.

In conclusion, this is a rare study that compares visual neuroadaptation in patients with two types of IOL implantations (Mu-IOL vs Mo-IOL) using resting-state fMRI analysis. Early postoperative neuroadaptation in patients with Mu-IOL implantation was observed. This study revealed different trends in the active levels of visual cortexes in patients with Mu-IOL and Mo-IOL implantations and provides a clinically significant reference for IOL selections before surgery.

## Data Availability Statement

The raw data supporting the conclusions of this article will be made available by the authors, without undue reservation.

## Ethics Statement

The studies involving human participants were reviewed and approved by the Ethics Committee of the ZOC at Sun Yat-sen University (2014MEKY035). The patients/participants provided their written informed consent to participate in this study.

## Author Contributions

HL, LZ, DL, and WCa contributed to data collection, analysis and interpretation, and manuscript preparation. LZ and DL co-wrote the first draft of the report. HL, WX, YZ, CC, XD, YX, YL, and YW critically revised the manuscript. LZ, HL, WCe, and YL obtained the research funding, coordinated the research and supervised the project. All authors reviewed the manuscript for important intellectual content and approved the final manuscript.

## Conflict of Interest

The authors declare that the research was conducted in the absence of any commercial or financial relationships that could be construed as a potential conflict of interest.

## Footnotes

1 http://rfmri.org

## Abbreviations

IOL: intraocular lens
Mu-IOL: multifocal IOL
Mo-IOL: monofocal IOL
ARCs: age-related cataracts
fALFF: fractional amplitude of low-frequency fluctuations
CS: contrast sensitivity
fMRI: functional magnetic resonance imaging
ZOC: Zhongshan Ophthalmic Center
VA: visual acuity
UCDVA: uncorrected distance visual acuity
BCDVA: best-corrected distance visual acuity
SV: straylight value
PVEP: pattern visual evoked potential
PVA: primary visual area
BA: Brodmann areas
RGCs: retinal ganglion cells.
