# Supplementary material for: Comparison of Visual Neuroadaptations After Multifocal and Monofocal Intraocular Lens Implantation
Source: Front Neurosci. 2021 Jun 14;15:648863. doi: 10.3389/fnins.2021.648863 (PMC8236945; doi:10.3389/fnins.2021.648863)
Supplement: Supplementary file 1 [file Data_Sheet_1.doc]

**Visual acuity, retinal straylight, contrast sensitivity, pattern visual evoked potential and fMRI examinations**

Distance visual acuity was examined with a Standard Logarithmic Visual Acuity E chart (Snellen). Uncorrected distance visual acuity (UCDVA; logMAR) and best-corrected distance visual acuity (BCDVA; logMAR) were recorded preoperatively and at 1 week, 3 months, and 6 months postoperatively.

The measurement of retinal straylight value (SV) was performed with a C-Quant straylight meter (Oculus Optikgerate GmbH, Wetzlar, Germany). The experimental setup was constructed by a series of concentric areas. The smallest area in the center was divided into two halves. Patients were asked to look at this test field while its concentric ring, the source of straylight, flickered with varying intensity to induce perceptible flicker in the test field. They were then asked to compare both halves of the test field, one of which had some counterphase flickers added and to press the button on either side that they perceived to have been flickering more intensively. The chosen sides defined a psychometric function from which the straylight value was obtained.

Contrast sensitivity (CS) was tested by Contrast Glare Tester 1000 (CGT-1000 Takagi Seiko Co. Ltd, Nagano, Japan) at 1 week, 3 months, and 6 months postoperatively. The test was conducted at a distance of 35 cm from the screen and took approximately 1 min per eye. The contrast threshold was presented in 12 levels from 0.01 to 0.45 and was determined in a graded stepwise manner similar to that used in automatic visual field tests. The patient was required to press a button when he or she observed a stimulus. The CS function was always measured in one’s best distance-corrected vision to prevent residual refractive error from affecting CS values. Absolute values of log CS were used in statistical analysis, and normalized values were used for graphical representation.

The pattern visual evoked potential (PVEP) was recorded using an Espion system (Diagnosys LLC, Lowell, MA, USA) as recommended by the International Society for Clinical Electrophysiology of Vision (ISCEV). All patients were instructed to remain fixated on the center of a stimulus located at a distance of 1 m in front of a 20 × 30 cm black-and-white video display monitor (contrast 99%). The reversal rate was 1 per second. The checkerboard stimulus subtended a visual angle of 5.7^0^ vertically and 8.5^0^ horizontally on either side of the fixation point. The P100 amplitude and latency for checkerboard sizes (15 min of arc) were recorded. An electrophysiology technician closely monitored the fixation stability of their eyes. If a patient was not cooperative, then the PVEP examination would be repeated for him or her. All PVEP examinations were performed in the same laboratory.

MRI acquisitions were performed on a MAGNETOM Verio 3T MR scanner (A Tim System; Siemens, Erlangen, Germany). Resting-state fMRI scans were performed using an echo-planar imaging sequence on the following settings: repetition time = 3,000 ms, echo time = 30 ms, flip angle = 90^0^, matrix = 64 × 64, field of view = 192 × 192 mm^2^, slice thickness = 3 mm, and slice gap = 0.5 mm. Each brain volume was composed of 41 axial slices, and each functional run contained 124 volumes. During the scans, all subjects were instructed to look forward through the center of the mirror (open the eye), fix their view in that direction and stay still with no thinking. The structural magnetization prepared a rapid gradient echo imaging sequence that was used to acquire structural T1-weighted images in the sagittal orientation. The following parameters were employed: repetition time = 1900 ms, echo time = 2.52 ms, flip angle = 9^0^, acquisition matrix = 246 × 256, and field of view = 250 × 250 mm^2^. The scanning time was 258 s, and a total of 176 1-mm-thick image slices were obtained. During the entire scan, subjects were instructed to keep their eyes open, relax and remain as motionless as possible. The fMRI images were preprocessed using the Statistical Parametric Mapping software package (SPM8; Welcome Trust Centre for Neuroimaging, University College London, London, UK; available in the public domain at http://www.fil.ion.ucl.ac.uk/spm).

**Data preprocessing and fALFF analysis**

Standard professional data processing software, Data Processing Assistant for Resting-State fMRI (DPARSF 2.2; State Key Laboratory of Cognitive Neuroscience and Learning, Beijing Normal University, Beijing, China; available in the public domain at <http://rfmri.org/DPARSF>), was used. DPARSF, the plug-in software running on a matrix laboratory platform (MATLAB R2013b; MathWorks, Inc., Natic, MA, USA), was based on statistical parametric mapping (SPM8; Welcome Trust Centre for Neuroimaging, University College London, London, UK; available in the public domain at http://www.fil.ion.ucl.ac.uk/spm) and a resting-state fMRI data analysis toolkit (REST 1.8; Song *et al*., available in the public domain at http://rfmri.org). The preprocessing steps were as follows: After DICOM files were converted to NIFTI images, the first 10 time points were discarded. Slice timing and spatial realignment were performed. A linear regression model was then used to remove the effects of head motion and other possible sources of artifacts, which included the Friston 24 head motion parameters, global mean signal, cerebrospinal fluid signal, and white matter signal. The remaining data were then normalized to the Montreal Neurological Institute (MNI) space using echo-planar imaging templates and resampled as 3-mm isotropic voxels. The linear timeline was removed. Finally, the fMRI-wave form of each voxel was temporally bandpass filtered (0.01–0.08 Hz).

The Resting-State fMRI Data Analysis Toolkit (http://rfmri.org) was used to calculate fALFF. The amplitudes of low-frequency fluctuations (ALFF) represent the intensity of low-frequency oscillations (LFOs), which reflect the activity of spontaneous neurons. Fractional ALFF (fALFF) is an index used to detect small differences, reflecting the relative contribution of specific LFOs to the entire range of oscillation/fluctuation frequencies, and it can be used as a primary parameter for the intensity of spontaneous visual cortical activity. The time series of each voxel was transformed to the frequency domain using a fast Fourier transform (FFT) (default setting: taper percent = 0, FFT length = shortest), and a power spectrum was obtained. The square root of this power spectrum was then averaged across each voxel in the 0.01 to 0.08 Hz frequency range, and this corresponding value was recorded as ALFF. fALFF is defined as the ratio of the ALFF value in a given frequency band (0.01-0.08) to the value of ALFF over the entire range of detectable frequencies in a given signal. This variable (fALFF) was defined as it might be more robust to physiological noise. Finally, all of the fALFF images were smoothed with a Gaussian filter/kernel with a 6-mm full-width half maximum.

**References**

Audoin, B., Fernando, K.T., Swanton, J.K., Thompson, A.J., Plant, G.T., and Miller, D.H. (2006). Selective magnetization transfer ratio decrease in the visual cortex following optic neuritis. *Brain* 129**,** 1031-1039.

Lin, H., Zhang, L., Lin, D., Chen, W., Zhu, Y., Chen, C., Chan, K.C., Liu, Y., and Chen, W. (2018). Visual Restoration after Cataract Surgery Promotes Functional and Structural Brain Recovery. *EBioMedicine* 30**,** 52-61.

Logothetis, N.K., Pauls, J., Augath, M., Trinath, T., and Oeltermann, A. (2001). Neurophysiological investigation of the basis of the fMRI signal. *Nature* 412**,** 150-157.

Odom, J.V., Bach, M., Brigell, M., Holder, G.E., Mcculloch, D.L., Mizota, A., and Tormene, A.P. (2016). ISCEV standard for clinical visual evoked potentials: (2016 update). *Doc Ophthalmol* 133**,** 1-9.

Puell, M.C., Benitez-Del-Castillo, J.M., Martinez-De-La-Casa, J., Sanchez-Ramos, C., Vico, E., Perez-Carrasco, M.J., Pedraza, C., and Del-Hierro, A. (2006). Contrast sensitivity and disability glare in patients with dry eye. *Acta Ophthalmol Scand* 84**,** 527-531.

Waberski, T.D., Gobbele, R., Lamberty, K., Buchner, H., Marshall, J.C., and Fink, G.R. (2008). Timing of visuo-spatial information processing: electrical source imaging related to line bisection judgements. *Neuropsychologia* 46**,** 1201-1210.
